# Mitophagy-related genes could facilitate the development of septic shock during immune infiltration

**DOI:** 10.1097/MD.0000000000035154

**Published:** 2023-10-20

**Authors:** Yu-Shen Yang, Wan-Jing Zheng, Chu-Yun Liu, Wei-Can Chen, Wen-Xi Xie, He-Fan He

**Affiliations:** a Department of Anesthesiology, The Second Affiliated Hospital of Fujian Medical University, Quanzhou, Fujian Province, China.

**Keywords:** diagnosis and prognosis, immune infiltration, integrative analysis, mitophagy, sepsis, septic shock

## Abstract

Septic shock often occurs following critically low blood pressure in patients with sepsis, and is accompanied by a high death rate. Although mitophagy is associated with infection and immune responses, its role in septic shock remains unknown. This study screened effective mitophagy-related genes (MRGs) for medical practice and depicted immune infiltration situations in patients with septic shock. Gene expression profiles of GSE131761 from the Gene Expression Omnibus database were compiled for differential analysis, weighted gene co-expression network analysis, and immune infiltration analysis, while other GSE series were used as validation datasets. A series of validation methods were used to verify the robustness of hub genes, while a nomogram and prognosis model were established for medical practice. Six genes were screened via combinations of differentially expressed genes, weighted gene co-expression network analysis, and MRGs. From this, 3 hub genes (MAP1LC3B, ULK1, and CDC37) were chosen for subsequent analysis based on different validation methods. Gene set enrichment analysis showed that leukocyte trans-endothelial migration and the p53 signaling pathway were abnormally activated during septic shock. Immune infiltration analysis indicated that the imbalance of neutrophils and CD4 naive T cells was significantly correlated with septic shock progression. A nomogram was generated based on MAP1LC3B, ULK1, and CDC37, as well as age. The stability of our model was confirmed using a calibration plot. Importantly, patients with septic shock with the 3 highly expressed hub genes displayed worse prognosis than did patients without septic shock. MAP1LC3B, ULK1, and CDC37 are considered hub MRGs in the development of septic shock and could represent promising diagnostic and prognostic biomarkers in blood tissue. The validated hub genes and immune infiltration pattern expand our knowledge on MRG functional mechanisms, which provides guidance and direction for the development of septic shock diagnostic and therapeutic markers.

## 1. Introduction

Sepsis is a life-threatening organ dysfunction syndrome commonly caused by a disproportionate host reaction to infection.^[1]^ Septic shock is regarded as a serious complication of sepsis characterized by metabolic and circulatory abnormalities as well as a high death rate.^[2]^ It has been reported that approximately 11 million patients died due to sepsis in 2017, accounting for nearly 20% of the global deaths.^[3]^ Importantly, an estimated 30% to 50% of the patients with septic shock die in hospitals,^[4]^ leading to considerable healthcare costs and burden.^[3]^ The overactive immune-inflammatory response triggered by infection has been identified to be a vital cause for sepsis-mediated extensive tissue damage and organ dysfunction.^[5]^ Thus, exploring the biological mechanisms underlying septic shock and the latent biomarkers involved in it is paramount to decreasing septic shock-related mortality.

Mitochondrial autophagy (mitophagy), a key cell viability which refers to selectively eliminating the excess or dysfunctional mitochondria via the specific mechanism of autophagy,^[6]^ has recently gained attention because of the protective role of mitophagy in numerous diseases, including sepsis.^[7]^ Impaired mitochondria play an important role in the induction of inflammation and organ dysfunction during sepsis through the overproduction of mitochondria-derived danger-associated molecular patterns and mitochondrial reactive oxygen species.^[8]^ Mitophagy can effectively reduce mitochondrial danger-associated molecular patterns and mitochondrial reactive oxygen species, thereby controlling inflammation.^[9]^ However, although the protective roles of mitophagy in several organs have been demonstrated during sepsis, mitophagy has been shown to have the opposite effect on skeletal muscle.^[7]^ It was reported that the inactivation of autophagic signaling by amino acid supplementation can inhibit sepsis-induced muscle protein degradation.^[10]^

Although mitophagy is closely associated with the organ function during sepsis, the possible correlation between mitophagy and septic shock, especially the potential role of mitophagy on immune cell activity during septic shock, remains unknown. Key biomarkers have been screened for disease diagnosis and prognosis evaluation using gene chip technology. Herein, we used the gene expression omnibus (GEO) database to identify the significance of blood mitophagy-related gene (MRG) expression levels between patients with and without septic shock. In addition, we investigated the role of immune cell infiltration and mitophagy on the development of septic shock, as well as their association with patient prognosis.

## 2. Materials and methods

### 2.1. Microarray dataset selection and acquisition

With “sepsis” or “septic shock” as the retrieval condition in the GEO database (https://www.ncbi.nlm.nih.gov/geo/), microarray datasets were selected and downloaded. Based on our retrieval condition, the GSE131761 series was extracted from the GEO database, which contained chips data of 129 whole blood samples from 15 patients (control), 33 patients without septic shock, and 81 patients with septic shock. Among them, blood samples from 33 patients without septic shock (as control group) and 81 patients with septic shock (septic shock group) were chosen for our subsequent differentially expressed genes (DEGs) and weighted gene co-expression network analyses (WGCNA). GSE137340, GSE8121, GSE65682, and GSE64457 series were also obtained from the GEO database and used for further validation in the current study and to reduce clinical bias among different studies. The data of this study are from public online databases (mainly from GEO database), and are not involved in patients rights and privacy. Thus, additional ethical approval from our hospital was unnecessary. The demographic information of all datasets in this study is shown in Table 1.

**Table 1:** Demographic information of all datasets.

Module name	Samples	Control	Septic shock	Platforms
GSE131761	Whole blood	33	81	GPL13497
GSE137340	Whole blood	12	6	GPL10558
GSE8121	Whole blood	15	30	GPL570
GSE65682	Whole blood	323	479	GPL13667
GSE64457	Purified neutrophils	8	15	GPL570

### 2.2. Gene expression profile preprocessing

We downloaded 1 type of file format for analysis “series_matrix_file” data (“.txt” file format), which contained GSE131761, GSE137340, GSE8121, GSE65682, and GSE64457. “R” software (R v4.2.1) was used for the analysis. First, the probe sets in the file formats were converted into gene symbols according to the manufacturer’s instructions for the bioconductor package. Next, we removed these probe sets without corresponding gene symbols and retained the mean expression value for different probe sets targeting the same gene. The robust multiarray averaging algorithm was used to normalize the data. Finally, the “limma” package (Limma package R 3.4.3) was used to screen DEGs. The adjusted *P* value < .05 and |fold change| > 1.2 were used to determine DEGs among these samples. The volcano plot was generated using the “ggplot” package.

### 2.3. Functional and pathway enrichment analysis

The DAVID database (https://david.ncifcrf.gov/) was used for functional enrichment analysis of DEGs. A common online platform (bioinformatics, http://www.bioinformatics.com.cn/) was used for the visual presentation of the functions and pathways including gene ontology (GO, consisting of biological process, cellular component, and molecular functional) terms and Kyoto encyclopedia of genes and genomes (KEGG) terms with a cutoff value of FDR < 0.05. The “clusterProfiler” package in R was used to perform the gene set enrichment analysis (GSEA)^[11]^ on the gene expression matrix with the aim to identify essential biological functions or pathways which may be ignored by differential analysis, based on all genes and phenotypes. The “c2.cp.kegg.v7.4.symbols.gmt” package was used to analyze significant enrichment between the control and septic shock groups. Subsequently, the results were illustrated in an enrichment plot using the “enrichplot” package. Gene size > 20, *P* value < .05 and |enrichment scores| > 0.4 were considered significant.

### 2.4. Evaluation of immune cell profiles among blood samples

A total of 22 types of immune cells were evaluated from blood tissues from GSE131761 to obtain comprehensive knowledge regarding immune cells infiltration. The official website (https://cibersort.stanford.edu/) was screened to download the “Cibersort.R” code and standard immune cell expression file “LM22.txt,” which were subsequently uploaded into R. Based on the gene expression profiles, the abundance of immune cell members from 22 mixed cells population were depicted, and proportion situation, correlation heatmap, as well as different expression levels between immune cells and samples were comprehensively analyzed and visually presented.

### 2.5. WGCNA

All genes from the GSE131761 dataset were used for WGCNA using the “WGCNA” package and its related packages in R. This was done to avoid erroneous results from the top genes according to variance. First, we performed hierarchical clustering analysis to exclude outliers from the included samples. To acquire the real biological network state (scale-free network), we calculated the soft threshold power and determined the optimal value for network construction.

On the basis of the most appropriate soft threshold power, we constructed the weighted gene co-expression network based on the links among gene expression. Subsequently, co-expression modules were determined and clustered using their similarity to each other. Each clustered module was set to have at least 30 genes. After calculating the adjacency of eigengenes via principal component analysis (PCA) dimensions and correlating them with each other, the correlations between clinical traits and modules were identified by calculating the module-trait Pearson correlations. Moreover, the module eigengenes and phenotypes were depicted using hierarchical clustering analysis.

Finally, the module membership and gene significance between each module and septic shock were calculated and displayed based on the identified correlations among phenotypes and these clustered modules. Finally, the modules with high correlation coefficients and cutoff values of *P* < .05 were selected as the key modules and used for further analysis.

### 2.6. Hub genes signature screening among MRGs

We identified 34 MRGs from the molecular signatures database^[12]^ and PathCards database (https://pathcards.genecards.org/). On the basis of the screening and identification of DEGs as well as key modules from the WGCNA, we assessed the correlations between MRGs and septic shock progression in blood samples. Finally, the “VennDiagram” package in R was used for the Venn plot analysis to identify and obtain the more accurate and convincing hub genes by choosing the intersection of 3 parts of genes.

### 2.7. Hub genes validation and PCA among different databases

PCA was used for dimension reduction of the hub genes to visualize whether they could be used to differentiate patients with and without septic shock (using the “prcomp” and “princomp” functions in R). The “scatterplot3d” package in R was used to display the results in a 3D-scatter plot. Further, the GSE series, GSE137340, and GSE8121, were used to identify the expression of hub genes and obtain hub genes in patients with septic shock more accurately.

### 2.8. Correlation analysis between hub genes and immune infiltration

Further, the validated genes were used to investigate the correlations with immune cell infiltrations. The GSE series GSE64457 was used for the validation analysis as it contained gene expression information regarding neutrophils in patients with sepsis and control patients.

### 2.9. Clinical diagnostic applications based on 3 hub MRGs

Receiver operating characteristic curves (ROC) were displayed and area under the curve (AUC) values were calculated to assess the predictive ability of each hub gene. Next, an innovative nomogram was constructed to allow for precise diagnosis during early stages of septic shock.

### 2.10. Survival curve analysis

After evaluating the hub genes diagnostic roles, we used the GSE65682 dataset to further assess the septic shock prognosis predictive function of the 3 hub genes. This dataset contained 479 patients with sepsis, with gene and prognosis data for each sample. The mean expression value of hub genes was set as the starting value of high expression. The patients survival odds were categorized into 4 grades according to the number of highly expressed hub genes: no risk (with no highly expressed hub genes), low risk (with 1 highly expressed hub genes), moderate risk (with 2 highly expressed hub genes), and high-risk (with 3 highly expressed hub genes).

## 3. Statistical analysis

R (version 4.1.2, using the different packages which mentioned above) and SPSS version 20.0 (IBM, Chicago, IL) were used to perform statistical analyses and visualization. Spearman correlation coefficient was used to evaluate the correlation between continuous variables. Statistical analysis between 2 groups was conducted using the Student *t* test, and for comparing 3 or more groups, 1-way analysis of variance was used. The survival curves were estimated using the Kaplan–Meier method. *P* values < .05 were considered statistically significant.

## 4. Results

### 4.1. Identification of DEGs and enrichment analysis in patients with septic shock

**Figure 1.**
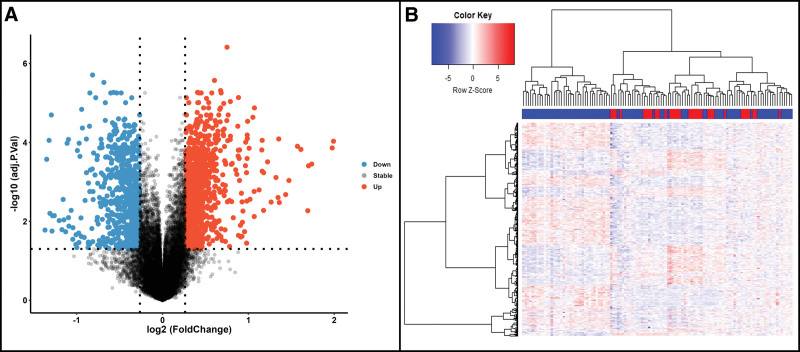
(A) Volcano plot of differentially expressed genes (DEGs), blue dots represent downregulated genes, red dots represent upregulated genes, and black dots represent normal genes. (B) Heatmap of DEGs in GSE131761 dataset.

**Figure 2.**
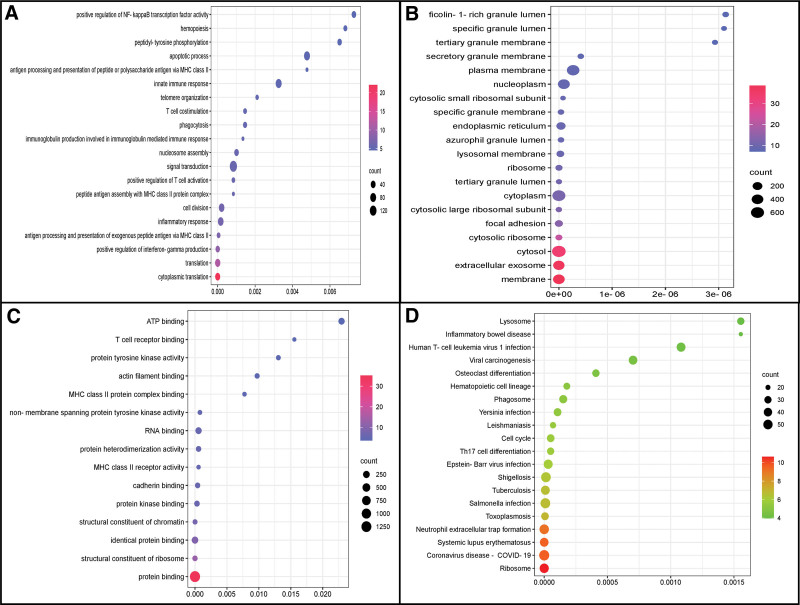
(A–C) Enrichment bubble plot illustrating the first 20 terms of biological processes and cellular components, as well as 16 molecular functional terms from Gene Ontology analysis. (D) Enrichment bubble plot illustrating the first 20 terms of Kyoto encyclopedia of genes and genomes analysis.

A total of 1820 genes were identified as DEGs, among which, 1063 were upregulated and 757 were downregulated. Volcano plot and a hierarchical clustering analysis was used to illustrate the distribution and expression of DEGs (Fig. 1A and B). Figure 2A–C displays the GO analysis results (see Table S1, Supplemental Digital Content, http://links.lww.com/MD/K393, which summarizes detailed results of GO analysis). The major variations in biological process were associated with antigen processing and presentation of exogenous peptide antigen via MHC class II, positive regulation of T cell activation, peptide antigen assembly with MHC class II protein complex, immunoglobulin production involved in immunoglobulin mediated immune response, T cell co-stimulation, and the innate immune response. Most differences in cellular component were observed at the membrane, extracellular exosome, cytosol, and cytosolic ribosome. The primary variations in molecular functional were associated with protein binding, structural constituent of ribosome, MHC class II receptor activity, and T cell receptor binding. Figure 2D shows the results of the KEGG analysis (see Table S2, Supplemental Digital Content, http://links.lww.com/MD/K394, which summarizes detailed results of KEGG analysis). It illustrates that DEGs obviously enriched in ribosome, COVID-19, systemic lupus erythematosus, neutrophil extracellular trap formation, toxoplasmosis, and salmonella infection.

**Figure 3.**
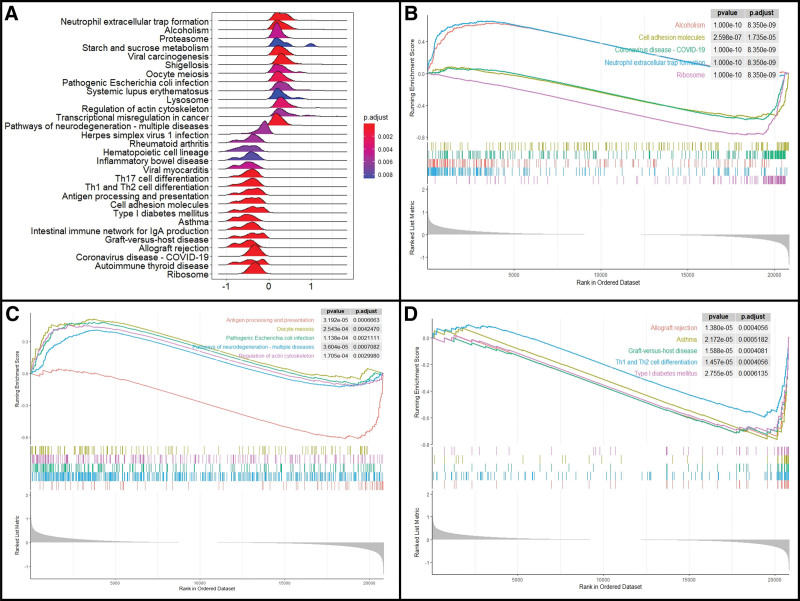
(A) Ridge plot, and (B–D) line graph of gene set enrichment analysis.

The genes included in the GSEA and the top fifteen significant results in the control and septic shock groups are illustrated in Figure 3A–D (see Table S3, Supplemental Digital Content, http://links.lww.com/MD/K395, which summarizes detailed results of GSEA analysis). The results indicate that ribosome, COVID-19, cell adhesion molecules, autoimmune thyroid disease, intestinal immune network for IgA production, and Th17 cell differentiation were enriched in the control group, while neutrophil extracellular trap formation, alcoholism, viral carcinogenesis, shigellosis, pathogenic *Escherichia coli* infection, leukocyte trans-endothelial migration, and p53 signaling pathway were enriched in patients with septic shock. In short, these results indicate that immune-related activity and pathways were significantly different between the 2 groups.

### 4.2. Immune landscape associated with venous blood from patients with septic shock

**Figure 4.**
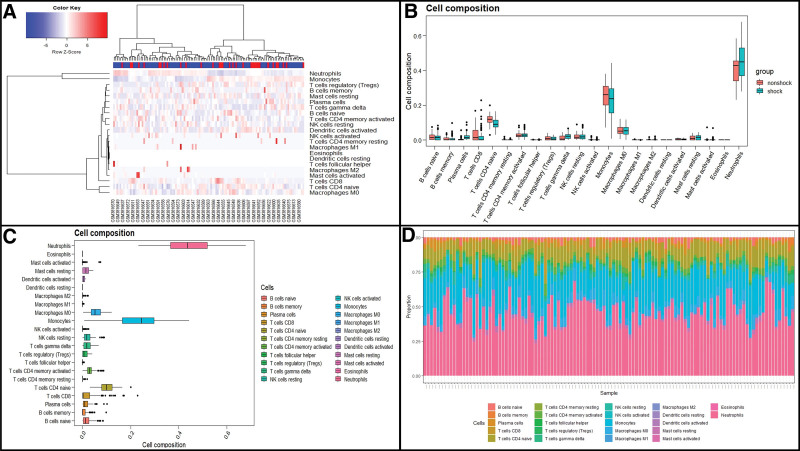
Analysis of immune landscape in blood tissue associated with septic shock. (A) Heatmap showing the distribution of 22 types of immune cells in blood of septic shock and control patients in GSE131761 dataset. (B) Boxplot illustrating the comparison of immune infiltration levels between patients with septic shock and control patients. (C) Boxplot illustrating the whole composition of immune cells in these patients. (D) Pile-up plot illustrating proportion of immune cells in each sample.

Next, the level of infiltrating immune cells in blood samples from septic shock and control groups was assessed using the CIBERSORT algorithm. The distribution of 22 immune cells in blood samples were clustered and presented in Figure 4A. We discovered that CD4 naive T cells were significantly downregulated in the blood of patients with septic shock (*P* = .003), whereas neutrophils were abnormally upregulated (*P* = .021) (Fig. 4B). Figure 4C–D illustrates the expression levels and proportion of immune cells in each sample, suggesting that the composition of neutrophils, monocytes, macrophages M0, and CD4 naive T cells were dominant in blood tissues, while eosinophils, activated mast cells, resting dendritic cells, M2 macrophages, M1 macrophages, activated natural killer cells, follicular helper T cells, and resting CD4 memory T cells were seldom observed in blood samples.

### 4.3. Construction of weighted gene co-expression network and key module identification of septic shock

**Figure 5.**
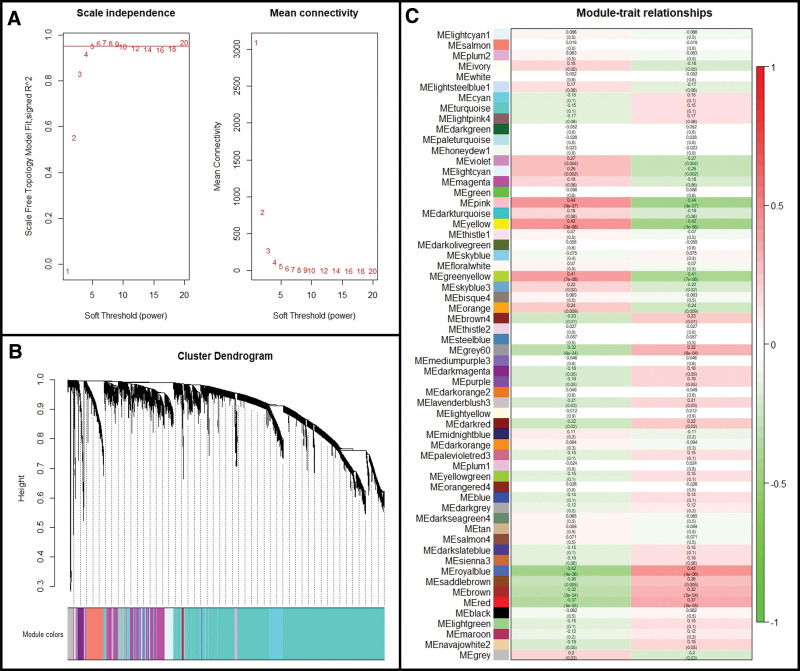
Weighted gene co-expression network analysis. (A) Evaluation of soft threshold power. Left panel indicates scale-free fit index, and right panel displays mean connectivity of these values. (B) Clustering dendrogram of all the genes based on dissimilarity measure (1-TOM) and assignment modules. (C) Module-trait relationships between different modules and features.

Further, the WGCNA algorithm was used to construct co-expression modules and confirm mRNA-related modules (see Table S4, Supplemental Digital Content, http://links.lww.com/MD/K396, which summarizes detailed results of WGCNA analysis). The soft threshold power was set to WGCNA to ensure the successful weighted gene co-expression network construction (Fig. 5A). Using the gene mutual co-expression levels, we conducted a hierarchical clustering tree analysis to cluster genes and generate modules which interacted with each other. We identified 60 co-expression modules (Fig. 5B). As shown in the column of septic shock group in Figure 5C, the pink (*R* = 0.44; *P* = 9e–07), yellow (*R* = 0.42; *P* = 3e–06), green-yellow (*R* = 0.41; *P* = 7e–06), and royal-blue (*R* = 0.42; *P* = 4e–06), etc modules showed strong correlations with septic shock progression (*P* < .05), and genes in these modules could facilitate or inhibit the development of septic shock. Thus, these modules were regarded as key modules (a total of 16 modules and 8965 genes) for further analysis and are summarized in Table 2.

**Table 2:** Basic characteristics of key modules correlated with septic shock.

Module name	Cor coefficient	*P* value	Gene number
MEviolet	0.27	.004	101
MElightcyan	0.29	.002	237
MEpink	0.44	9e-07	739
MEyellow	0.42	3e-06	1318
MEgreenyellow	0.41	7e-06	416
MEskyblue3	0.22	.02	71
MEorange	0.24	.009	180
MEbrown4	−0.23	.01	50
MEgrey60	−0.32	6e-04	236
MElavenderblush3	−0.21	.03	34
MEdarkred	−0.22	.02	211
MEroyalblue	−0.42	4e-06	219
Me saddle brown	−0.26	.005	112
MEbrown	−0.32	5e-04	1318
MEred	−0.37	5e-05	1132
MEgrey	0.2	.03	2591

### 4.4. Selection and validation of MRGs in septic shock progression

**Figure 6.**
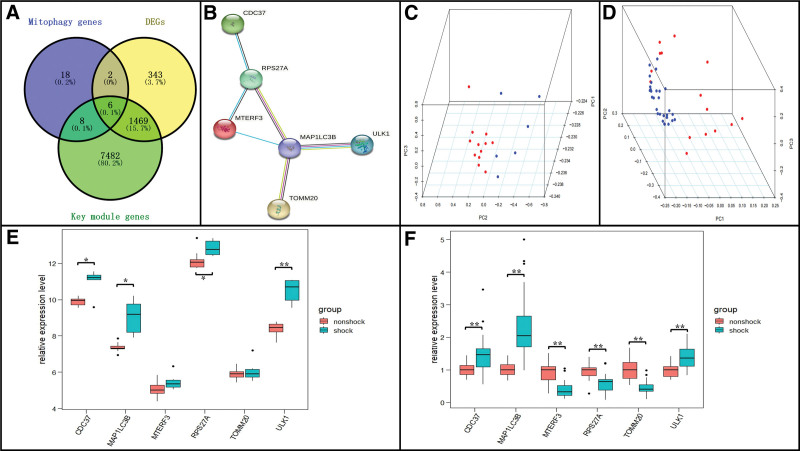
(A) Venn diagram indicating six genes were commonly expressed in three parts. (B) The protein-protein interaction network was constructed to identify the hub genes. (C and D) 3D-scatter plot after principal component analysis dimension reduction of these six genes in GSE137340 and GSE8121 datasets. (E and F) Expression validation of these six genes in GSE137340 and GSE8121 datasets. Statistical significance is denoted by asterisks (*P < .05; **P < .001).

As previously mentioned, we showed that the expression levels of genes in key modules increased with the septic shock progression, and that 1820 mutual DEGs were highly correlated with septic shock. Venn diagrams were used to intersect the 1820 DEGs, 8965 genes in key modules, and 34 MRGs obtained from the online database. A total of 6 genes were commonly expressed in these 3 components of our Venn diagram, namely MAP1LC3B (upregulated), TOMM20 (upregulated), ULK1 (upregulated), CDC37 (upregulated), MTERF3 (upregulated), and RPS27A (downregulated) (Fig. 6A). Further, these genes were integrated in the protein-protein interaction network analysis (Fig. 6B).

To validate the robustness of these 6 genes, we performed a PCA between different databases. The dimension of these 6 genes was reduced into 3 principal components (PC1, PC2, and PC3), thus illustrating the clustering properties and the spatial distribution of data among septic shock and control groups. Our results further suggest that the 3 primary components could clearly distinguish the control samples from septic shock samples in a 3D system (Fig. 6C, D) after dimension decrease of these 6 genes from GSE137340 and GSE8121, respectively. Next, the expression levels of these 6 genes were identified in the GSE137340 and GSE8121 datasets, which included gene expression levels from blood samples from patients with septic shock and those from control patients. As shown in Figure 6E–F, the expression levels of MAP1LC3B, ULK1, and CDC37 were significantly upregulated in patients with septic shock compared to those in control patients. Thus, MAP1LC3B, ULK1, and CDC37 were considered hub genes and used in subsequent analyses. Although RPS27A was also significantly dysregulated in patients with septic shock compared to the levels observed in control patients (in both datasets), its expression in the GSE8121 dataset was opposite to that in the GSE137340 and GSE131761 datasets. Hence, RPS27A was excluded from further analysis.

### 4.5. Immune infiltration analysis in septic shock based on the 3 hub genes

**Figure 7.**
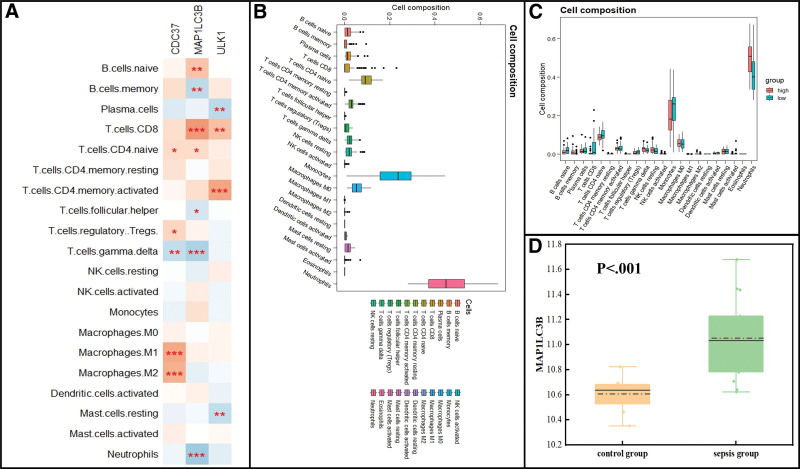
(A) Correlation analysis between MAP1LC3B, ULK1, CDC37, and immune infiltration in GSE131761 dataset. Statistical significance is denoted by asterisks (***P < .001; **P < .01; *P < .05). (B) Relationship of immune infiltration levels between MAP1LC3B-high and MAP1LC3B-low group in patients with septic shock. (C) Boxplot illustrating the whole composition of immune cells in patients with septic shock. (D) Further validation of MAP1LC3B expression in other datasets containing the gene expression information of neutrophils in sepsis.

As we showed that immune cells are closely correlated with the development of septic shock, the correlation between hub MRGs and immune infiltration was further investigated in the GSE131761 dataset. The correlation heatmap shows that MAP1LC3B has the highest correlation with MRG during immune cells (especially neutrophil) infiltration in septic shock (Fig. 7A). Subsequently, to reveal the potential association between MAP1LC3B expression and immune infiltration in the progression of septic shock, we divided the data from patients with septic shock (GSE131761) into 2 groups based on the mean expression levels of MAP1LC3B: low-MAP1LC3B group and high-MAP1LC3B group. The expression levels and proportion of immune cells in MAP1LC3B-related groups from septic shock was evaluated first. As shown in Figure 7B, our results indicate that in blood samples of patients with septic shock, neutrophils, monocytes, CD4 naive T cells, and M0 macrophages were dominant, while eosinophils, resting dendritic cells, and M1 macrophages were seldom present. Furthermore, immune results illustrated that the infiltration levels of neutrophils were positively associated with MAP1LC3B expression (Fig. 7C). To further verify the association between MAP1LC3B expression and neutrophils infiltration during sepsis, we used the GSE64457 dataset for the validation analysis as it contains neutrophil gene expression information for patients with sepsis and control patients. Our results indicate that MAP1LC3B was significantly upregulated in the neutrophil of patients with sepsis when compared to that in control patients (Fig. 7D).

### 4.6. Construction of a nomogram to predict septic shock

**Figure 8.**
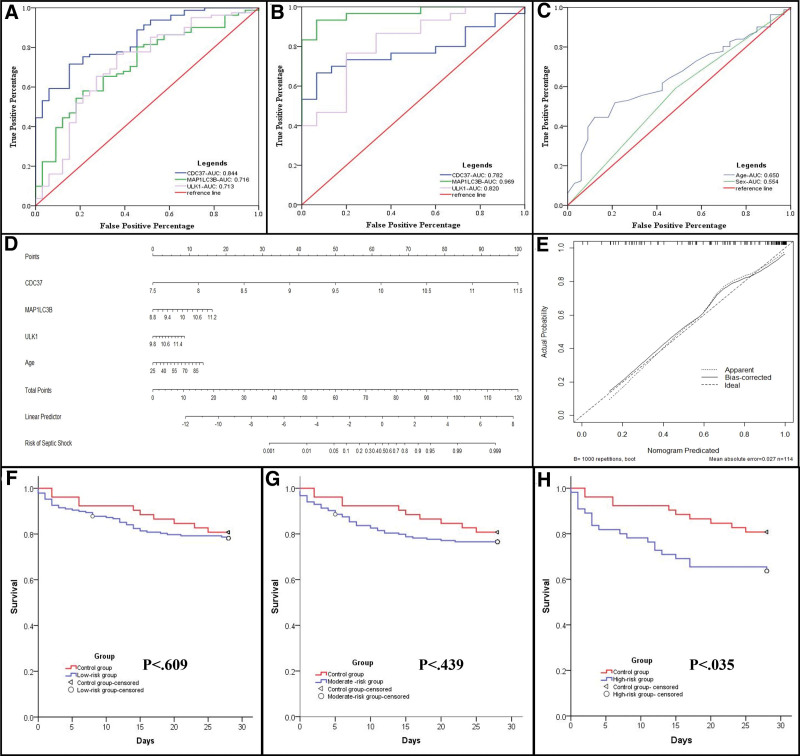
(A–C) ROC curve analysis of the three hub MRGs and clinical traits (age and sex) for predicting septic shock in different datasets. (D) Establishment of nomogram model based on three hub MRGs and clinical trait age. (E) Calibration plot of nomogram for evaluating robustness of the prediction. (F–H) Survival analysis. MRG = mitophagy-related gene, ROC = receiver operating characteristic curves.

Next, an ROC curve analysis was conducted to investigate the predictive value of each feature, including MAP1LC3B, ULK1, CDC37, gender, and age (Fig. 8A–C). Using the data from the GSE131761 and GSE8121 datasets, we determined that the AUC of MAP1LC3B was 0.716 and 0.969; that of ULK1 was 0.713 and 0.820; and that of CDC37 was 0.844 and 0.782, respectively. Next, a nomogram model was constructed to predict septic shock progression by combining a traditional clinical feature (age) with the expression levels of the 3 hub MRGs (Fig. 8D). The nomogram performance was visualized assessed using calibration plots, and the results indicated the ability of our model to predict septic shock outcomes (Fig. 8E). Taken together, our nomogram model indicates that higher age and higher expression of MAP1LC3B, ULK1, as well as CDC37 in blood samples could lead to a higher probability of septic shock initiation.

### 4.7. Survival curve analysis

Ultimately, the predictive value of the 3 hub MRGs for the prognosis of patients with septic shock was evaluated via Kaplan–Meier analysis. Among the 479 patients with sepsis from the GSE65682 dataset, 52 cases (10.9%) were categorized as no risk (control group), 188 (39.2%) were categorized as low risk, 184 (38.4%) were categorized as moderate risk, and 55 (11.5%) cases were categorized as high-risk. The patients with low and moderate risk did not show worse prognosis when compared to the control group (Fig. 8F, G). However, in the high-risk group, the prognosis of patients was significantly worse than that of patients in the control group (Fig. 8H).

## 5. Discussion

Sepsis is a serious public health problem worldwide and is associated with a high mortality rate. Septic shock is a life-threatening complication of sepsis and has a low survival rate. Especially during the COVID-19 pandemic, some severe and critically ill patients who displayed multiple-organ dysfunctions were diagnosed with sepsis, leading to increasing death rates.^[13]^ Early diagnosis and treatment are an effective and essential method to decrease the mortality and improve the prognosis of patients with septic shock. With the rapid development of molecular biology techniques, several useful biomarkers and measures were used to reveal the potential mechanisms of septic shock, thereby providing insight into the diagnosis, evaluation, and treatment. Herein, a bioinformatics analysis was performed to illustrate the potential biomarkers of septic shock using associated microarray datasets containing sepsis and septic shock data.

In the present study, a total of 1820 DEGs were screened, of which, 1063 were upregulated and 757 were downregulated in the septic shock samples compared to those in the controls. GO analysis illustrates that these DEGs were mainly involved in positive regulation of T cell activation, peptide antigen assembly with MHC class II protein complex, T cell co-stimulation, and innate immune responses, thereby indicating that the functions of DEGs were mainly enriched in immune-associated activities, and these immune functions were strongly related to septic shock. The results from KEGG enrichment analysis suggest that several dysfunctional signaling pathways correlated with these DEGs were observed in ribosome, COVID-19, systemic lupus erythematosus, neutrophil extracellular trap formation, toxoplasmosis, and salmonella infection. Consequently, we demonstrated that the aberrant alterations of immune responses as well as signaling pathways in blood samples of patients with septic shock occurred in the early phase of septic shock and can be thought as an early warning in the development of septic shock.

Next, the identified genes were investigated by GSEA to determine other signaling pathways that are fundamental but were ignored by the DEGs. In addition to the aforementioned signaling pathways, the GSEA results indicate that leukocyte trans-endothelial migration and the p53 signaling pathway were also aberrantly activated in septic shock. Furthermore, p53 was associated with immune cell survival and its overexpression is known to promote immunogenic cell death.^[14]^ Hence, the relationships between septic shock and immune functions were further supported by our GSEA analysis. In addition, WGCNA generated 60 different clustered co-expression modules, among which 16 modules displayed high correlations (*P* value < .05), and were validated as key modules in the progression of septic shock.

Subsequently, we depicted the blood-related immune landscape associated in patients with septic shock based on the identified DEGs that were strongly correlated with immune dysfunctions during the development of septic shock. The results indicate that the dysregulation of CD4 naïve T cells and neutrophils was obviously associated with the progression of septic shock. Neutrophils are the predominant immune cell population and play key roles in the innate immune system.^[15]^ As the first line of defense against pathogens, they are involved in the pathophysiology of several disease conditions.^[16,17]^ Activated neutrophils, on 1 hand, mainly utilize a series of effector mechanisms to protect against invading pathogens, while on the other hand, they also release various pro-inflammatory cytokines and chemokines to recruit other immune cells to the site of infection. Recently, neutrophil extracellular traps formation was regarded as an important mechanism against pathogens.^[18]^ In this study, we discovered that neutrophils were significantly upregulated in patients with septic shock, which is in line with previous findings. Uhel et al^[19]^ showed that neutrophil were significantly and specifically expanded in patients with sepsis when compared to control patients without sepsis. However, such neutrophils represent a “double-edged sword” in sepsis because of their pro-inflammatory and anti-inflammatory role.^[20]^ A previous study indicated that neutrophils are largely expanded and enriched during the early phase of the sepsis response, contributing to the eradication of microbes but also leading to the pathogenesis of sepsis.^[21]^ For example, neutrophils can regulate immunosuppression through genetic behavior (kinases), molecular alterations (cytokines, chemokines, and signaling mediators), and cellular alterations (cell–cell contact).^[22–24]^ In addition, aberrant neutrophil cell death in the later phase of sepsis is similarly hazardous because of the decreased body defenses and enhanced vulnerability to infections. These findings highlight the importance of neutrophils in mediating the development of septic shock. Our results also illustrate that CD4 naïve T cells were obviously dysregulated in patients with septic shock. CD4 naïve T cells are a primary subset of T lymphocytes which can differentiate into different functional subtypes (such as T helper types 1 and 2) and then exert important immunity functions.^[25]^ The lack of CD4 naïve T cells is highly correlated with immunodeficiency diseases.^[26]^ While the roles of CD4 naïve T cells in the development of septic shock have not been previously reported, we discovered that CD4 naïve T cells were significantly downregulated in septic shock. However, further research investigating the interactions between CD4 naïve T cells and septic shock is required.

Three MRGs (MAP1LC3B, ULK1, and CDC37) were obviously upregulated in blood samples of patients with septic shock, which were regarded as hub MRGs for septic shock. The fundamental relationship between mitophagy and immune infiltration has been reported by several studies.^[24,27]^ Subsequently, mitophagy was indicated to be strongly correlated with sepsis-induced organ dysfunction.^[28]^ These findings highlight the importance of mitophagy in sepsis. However, whether mitophagy is involved in the development of septic shock has not been reported. This study provides evidence for the relationship between the 3 hub MRGs and septic shock. We investigated the correlations between the 3 MRGs and immune infiltration during septic shock and discovered that MAP1LC3B was both significantly correlated with CD4 naïve T cells and neutrophils infiltration. Thus, MAP1LC3B was considered the most correlated MRGs with immune cells (especially neutrophil) infiltration during septic shock. Then, immune infiltration analyses of septic shock patient data were further performed considering the expression levels of MAP1LC3B. The results indicate that neutrophils infiltrations were increased in the MAP1LC3B-high group, which is consistent with our observations. To further reveal the relationship between MAP1LC3B and neutrophils infiltrations, other validation GSE series containing gene expression information regarding neutrophil in sepsis were included in our study. We discovered that MAP1LC3B was significantly upregulated in the neutrophils of patients with sepsis when compared to control patients. Taken together, these results confirm that patients with septic shock with high expression levels of MAP1LC3B were subject to a mediate immune response, thus making it easier to develop cascades of reactions that facilitate septic shock.

MAP1LC3B is one of the crucial autophagy-related proteins and its lipidated form is considered a biomarker for the autophagosomes.^[29,30]^ MAP1LC3B exerts its effects mainly by interacting with sequestosome 1, a substrate for mitophagy that transports the cargo into the autophagosomes for degradation.^[31]^ Various studies have investigated the core roles of MAP1LC3B in different diseases such as hepatitis C virus infection,^[32]^ oral squamous cell carcinoma,^[33]^ and Hermansky-Pudlak syndrome^[34]^; thus, it is considered a potential diagnostic and prognostic biomarker for a variety of patients. In addition, the association between MAP1LC3B and sepsis has been increasingly revealed in recent years. A study published by Klara Giegerich et al^[35]^ in 2014 observed a significant accumulation of MAP1LC3B-II in lipopolysaccharide-stimulated Raw264.7 macrophages and demonstrated the existence of a colocalized relationship between MAP1LC3B and pellino E3 ubiquitin protein ligase family member 3 (a pathway protein in the TLR signaling cascade). Two additional studies published recently also indicated that MAP1LC3B may influence the development of sepsis and sepsis-related acute kidney injury.^[36,37]^ Therefore, based on previous reports and the results of our study, we hypothesize that MAP1LC3B might have an important role in stimulating immune infiltration in blood tissues and thus contributes to the progression of septic shock. Currently, the specific relationships between MAP1LC3B and septic shock have not been revealed; thus, our findings provide reliable evidence for the correlation between the 2 and validate the fundamental roles of MAP1LC3B in septic shock-related immune infiltration.

ULK1 is an important element of the autophagy process and was identified to be involved in sepsis via various mechanisms. For instance, Zhang et al^[38]^ indicated that the acetylation of ULK1 RNA involves the NAT10-mediated neutrophil pyroptosis in sepsis. In addition, ULK1-dependent autophagy also plays a role in the protective effect of Sestrin 2 on sepsis-associated encephalopathy.^[39]^ These findings demonstrate the vital function of ULK1 in sepsis. Notably, it has also been validated that ULK1 activation is associated with another hub gene, CDC37, which usually interacts with a variety of kinases and, in cooperation with HSP90, directs the maturation of numerous morbigenous clients.^[40,41]^ In 2011, Hyuck Joo et al^[42]^ demonstrated that the interaction between ULK1 and Hsp90-CDC37 can stabilize and activate ULK1, which in turn is required for the phosphorylation and release of Atg13 from ULK1, as well as for the recruitment of Atg13 to damaged mitochondria. However, evidence on the association of ULK1 with sepsis is scarce. In this study, we demonstrated that ULK1 and CDC37 are closely related to septic shock-related immune infiltration, which provides new directions for designing the intervention and treatment of septic shock.

Furthermore, we investigated the clinical application value of these 3 hub MRGs. We first evaluated the predictive ability of each hub MRGs using the ROC curve and observed that the AUC of each ROC was > 0.7, suggesting a high predictive results of each hub MRG. Next, to improve the diagnosis precision during the early phase of septic shock, the 3 hub MRGs in blood tissue were combined with the patient age, as a clinical feature, to construct a nomogram model. The reliability of our model was confirmed using the calibration plot and indicting its feasibility for future clinical use. Finally, we evaluated the prognostic significance of 3 hub MRGs genes in septic shock using Kaplan–Meier analysis. The results illustrated that patients from the high-risk group displayed a worse prognosis compared to those in the control group. Consequently, high expression of MAP1LC3B, ULK1, and CDC37 in blood tissue could act as diagnostic and prognosis factors of septic shock.

Nevertheless, our study has several limitations. For instance, we used bioinformatics analyses of data from the GEO database, which could be different from actual scenarios. Furthermore, the data in the datasets did not include sufficient baseline information of patients, rendering it difficult to include various general information in our analysis. Moreover, for certain analyses, we set the mean expression of MAP1LC3B as the cutoff value to perform grouping in the absence of clinical data, which is undoubtedly biased. Ultimately, the relationships between clinical manifestations and gene expression remain to be further clarified.

Taken together, 1820 DEGs were selected following the differential analysis, 8965 genes in key modules were validated through the WGCNA, and ultimately, 6 common genes were identified as DEGs, key modules, as well as MRGs. MAP1LC3B, ULK1, and CDC37 were further considered as hub MRGs, diagnostic biomarkers, and prognostic factors in the development of septic shock, which were validated using the PCA and expression validation in other datasets. Subsequently, for early diagnosis of septic shock through the detection of these genes in blood samples, a nomogram model was constructed by combining the 3 hub MRGs with the patient’s age. Finally, a prognostic model was established to validate the predictive value of these hub MRGs in septic shock.

## Author contributions

**Conceptualization:** Yu-Shen Yang.

**Funding acquisition:** He-fan He.

**Methodology:** Wan-Jing Zheng.

**Supervision:** Wen-Xi Xie.

**Visualization:** Chu-Yun Liu, Wei-Can Chen.

**Writing – original draft:** Yu-Shen Yang, Wan-Jing Zheng.

**Writing – review & editing:** Wen-Xi Xie, He-fan He.

## Supplementary Material








